# 4-Chlorophenol Removal Using BiFeO_3_/MoS_2_ Piezoelectric Photocatalytic Material Coupled with Peroxymonosulfate

**DOI:** 10.3390/molecules31172987

**Published:** 2026-08-26

**Authors:** Huan Deng, Qingsong Xie, Shengnan Li, Hai Lu, Hongyan Wei, Tiehong Song

**Affiliations:** 1 School of Life Sciences, Changchun Sci-Tech University, Changchun 130600, China; 100343@cstu.edu.cn; 2 Key Laboratory of Songliao Aquatic Environment, Ministry of Education, Jilin Jianzhu University, Changchun 130118, China; xieqingsong@jlju.edu.cn (Q.X.); lishengnan@neau.edu.cn (S.L.); 3 Urban Construction College, Changchun University of Architecture and Civil Engineering, Changchun 130607, China

**Keywords:** BiFeO_3_/MoS_2_ composite, piezophotocatalytic system, peroxymonosulfate, 4-chlorophenol, mechanical stirring

## Abstract

In this work, a BiFeO_3_/MoS_2_ (BM) material was utilized to establish a piezophotocatalytic system under combined visible-light (Vis) illumination and mechanical stirring (MS), which synergistically activated peroxymonosulfate (PMS) toward the oxidation of 4-chlorophenol (4-CP), a representative refractory organic pollutant in water. Under mechanical stirring, the piezoelectric effect in BM generates a polarized electric field that promotes the separation of photogenerated electron–hole pairs, thereby providing more charge carriers for PMS activation and subsequent radical generation. The degradation performance, underlying mechanism, toxicity of degradation products, reusability, and applicability in different water matrices in the BM(1:3)/PMS process were comprehensively evaluated. The results indicated that BM(1:3) exhibited superior performance over other BM ratios (BM(2:1), BM(1:1), and BM(1:2)), achieving 91.6% removal of 4-CP within 30 min at a rotation speed = 1000 rpm, PMS = 2.0 mM, BM(1:3) = 0.5 mg/L, and initial 4-CP = 10 mg/L. Furthermore, water quality parameters exerted notable impacts on 4-CP decomposition. A pH of 4.7 was favorable, and the presence of Cl^−^ enhanced the 4-CP degradation, while HCO_3_^−^ and H_2_PO_4_^−^ suppressed the removal efficiency; SO_4_^2−^ and HA showed negligible influence. DFT calculations identified the reactive sites on 4-CP that are prone to attack by various reactive oxygen species (e.g., •O_2_^−^, ^1^O_2_, •OH, and SO_4_•^−^), resulting in the transformation of 4-CP through three degradation pathways into smaller organic intermediates, most of which were less toxic than 4-CP. The BM(1:3) catalyst exhibited satisfactory reusability; however, a significant decrease in 4-CP degradation efficiency was observed in the lake water matrix. Overall, the synergy between photocatalysis and the piezoelectric effect in the BM(1:3)/PMS system offers a useful research foundation for the piezophotocatalytic degradation of persistent organic pollutants such as 4-CP.

## 1. Introduction

The rapid expansion of the chemical industry has led to the widespread occurrence of emerging pollutants in water systems. Among these, 4-chlorophenol (4-CP) is one of the most commonly detected pollutants in various water sources. Even at trace concentrations, 4-CP exhibits biological toxicity and “teratogenic, carcinogenic, and mutagenic” effects (causing birth defects, cancer, and mutations), posing long-term risks to both aquatic life and human populations [1]. Due to its stable aromatic ring structure and large molecule size, 4-CP is resistant to biodegradation. Conventional biological treatment processes show limited removal efficiency for 4-CP; therefore, it is imperative to develop efficient advanced treatment technologies.

Advanced oxidation processes (AOPs) are capable of generating reactive oxygen species (ROS), which exhibit higher oxidation capacity than precursor oxidants and exert strong destructive effects on poorly biodegradable organic compounds. Consequently, various AOPs have attracted considerable attention and have been actively developed. Among these, photocatalytic oxidation utilizes light irradiation to activate semiconductor materials for the generation of electron–hole pairs, thereby producing reactive species like superoxide radicals (•O_2_^−^), hydroxyl radicals (•OH) and singlet oxygen (^1^O_2_), enabling the mineralization of most organic pollutants [2]. However, the light energy utilization efficiency of photocatalytic technology remains low, and photogenerated carriers are highly susceptible to recombination [3]. To overcome these drawbacks, the piezoelectric effect has been introduced. Piezoelectric materials generate a polarized electric field under mechanical stress (e.g., from stirring), which acts as a driving force to separate photogenerated carriers [4,5]. Common examples include perovskite oxides (e.g., BiFeO_3_) and layered dichalcogenides (e.g., MoS_2_). Coupling photocatalysis with piezoelectric catalysis to construct a piezo–photocatalytic synergy system can effectively overcome the limitations of either standalone photocatalysis or piezoelectric catalysis [6]. On this basis, further integrating the piezo–photocatalytic system with the oxidant activation, that is, utilizing the electrons and holes generated during the piezo–photocatalytic process to activate peroxydisulfate [7], peroxymonosulfate [8], hydrogen peroxide [9], and sodium percarbonate [10], enables the production of multiple highly reactive species including sulfate radicals (SO_4_•^−^), •OH, and carbonate radical (CO_3_•^−^). This establishes a promising strategy for further enhancing the mineralization efficiency.

Compared with various oxidants, peroxymonosulfate (PMS) is considered a highly promising oxidant due to its high oxidation-reduction potential and the ability to generate both SO_4_•^−^ and •OH upon activation, with SO_4_•^−^ exhibiting an extended half-life (30–40 µs) and broader pH tolerance than •OH [11]. Recent studies show that various oxidants like peracetic acid and persulfate can be activated by catalysts [12,13]. Here, PMS was specifically chosen because its asymmetric structure makes it more susceptible to activation via electron transfer from the photo- and piezo-generated charge carriers [14]. By utilizing piezoelectric–photocatalytic materials to synergistically activate PMS, one can not only achieve efficient PMS activation but also avoid secondary pollution caused by external chemical reagents [15]. Therefore, the exploration and fabrication of functional materials that possess excellent piezoelectric properties and photocatalytic activity, along with their application in activating PMS for the removal of organic pollutants in water, hold significant research value.

The synthesis of piezoelectric–photocatalytic materials is crucial for coupling with the PMS system in catalytic reactions. Over the past few years, numerous piezoelectric–photocatalytic materials have been developed for application in organic pollutant degradation. As an example, Li et al. [16] synthesized a BaTiO_3_/TiO_2_ nanocomposite via hydrothermal and topochemical reactions and studied its performance in photocatalysis and pressure-induced catalysis toward the removal of methyl orange (MO). Lv et al. [17] prepared MoS_2_/PVDF nanofibrous films and investigated their effect on activating PMS toward the removal of tetracycline. Recently, Zhou et al. [18] developed a ternary MoS_2_/Bi_2_S_3_/BiFeO_3_ heterojunction for ciprofloxacin degradation. While ternary systems show excellent performance, their synthesis is more complex. In this work, we chose a simpler binary BiFeO_3_/MoS_2_ system, which our previous study has shown to be effective for PMS activation [19]. This binary system provides a simpler platform to systematically investigate the fundamental mechanism of piezo–photocatalytic PMS activation for 4-CP degradation. Additionally, other newly developed synthetic materials such as α-SnWO_4_/ZnO and BiVO_4_/NaNbO_3_ have also been used for PMS activation in removing organic pollutants [20,21]. In our earlier work [19], we successfully synthesized BiFeO_3_/MoS_2_ and applied it for PMS activation in removing sulfamethoxazole (SMX), achieving relatively satisfactory degradation results. Building upon the above research foundation, this work further extends the application of BiFeO_3_/MoS_2_ to the catalytic degradation of 4-CP, with a systematic investigation of the removal performance and degradation mechanism of the BiFeO_3_/MoS_2_/PMS system toward 4-CP, aiming to offer a theoretical basis and technical reference for the efficient treatment of emerging pollutants.

## 2. Results and Discussion

### 2.1. Performance of Various Catalysts in Removing 4-CP

**Figure 1:**
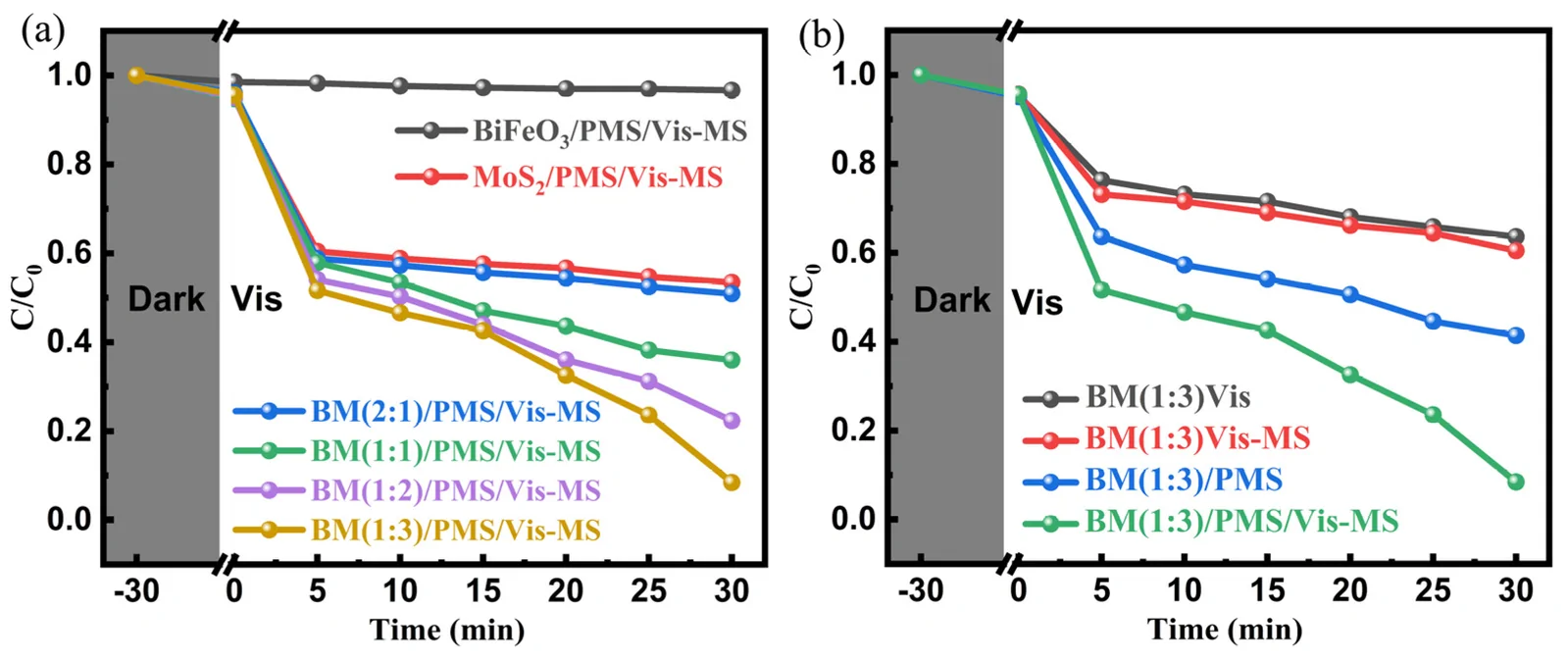
4-CP removal using different catalysts (**a**) and in various systems (**b**). Conditions: PMS = 2.0 mM, 4-CP = 10 mg/L, catalyst = 0.5 g/L, pH = 4.7, rotation speed = 1000 r/min, Xenon lamp (solar simulator, 300 W).

As depicted in Figure 1a, in the absence of light (−30 to 0 min), none of the materials (BiFeO_3_, MoS_2_, BM(2:1), BM(1:1), BM(1:2), and BM(1:3)) exhibited significant removal of 4-CP, indicating that these systems exhibit virtually no catalytic activity under dark conditions. Upon exposure to visible light, pure BiFeO_3_ exhibited only a very weak removal effect on 4-CP, with a removal rate of just 4.7% within 30 min. This indicates that it has virtually no piezoelectric photocatalytic activity under the tested conditions, which may be ascribed to its limited visible light response and rapid carrier recombination rate [22]. In contrast, pure MoS_2_ removed 46.7% of 4-CP within 30 min under visible light. This is attributed to MoS_2_’s excellent visible light absorption capacity, which enables it to generate a certain amount of electron–hole pairs upon light excitation, together with the abundant active sites provided by its layered structure, thereby promoting the oxidation of 4-CP [23,24,25]. Under identical conditions, the 4-CP removal efficiencies achieved by the composite catalysts BM(2:1), BM(1:1), BM(1:2), and BM(1:3) were 49.1%, 64.1%, 77.7%, and 91.6%, respectively. These findings demonstrate that the composites outperform the individual materials (BiFeO_3_, MoS_2_), and that the catalytic performance of the composites gradually improves with increasing MoS_2_ content. Among them, BM(1:3) exhibited the highest performance in activating PMS to degrade 4-CP. Figure 1b compares the capacity of BM(1:3) for 4-CP removal under different conditions (Vis only, MS only, Vis-MS, and Vis-MS/PMS). The removal efficiencies of 4-CP achieved by BM(1:3)/Vis, BM(1:3)/Vis-MS, BM(1:3)/PMS, and BM(1:3)/PMS/Vis-MS were 36.4%, 39.5%, 58.7%, and 91.6%, respectively. These observations show that the bare catalyst exhibits limited catalytic ability without an oxidant; however, upon introduction of PMS, the system’s degradation capability toward 4-CP is significantly enhanced. Furthermore, the total organic carbon (TOC) removal rates in the BM(1:3)/PMS/Vis and BM(1:3)/PMS/Vis-MS processes reached 30.5% and 45.2%, respectively, reflecting the mineralization of 4-CP. The superior ability of BM(1:3)/PMS/Vis-MS observed herein is consistent with our earlier study on SMX removal, further confirming that BM(1:3) is the most promising catalyst among the tested formulations [19].

### 2.2. Factors Influencing the Removal of 4-CP

**Figure 2:**
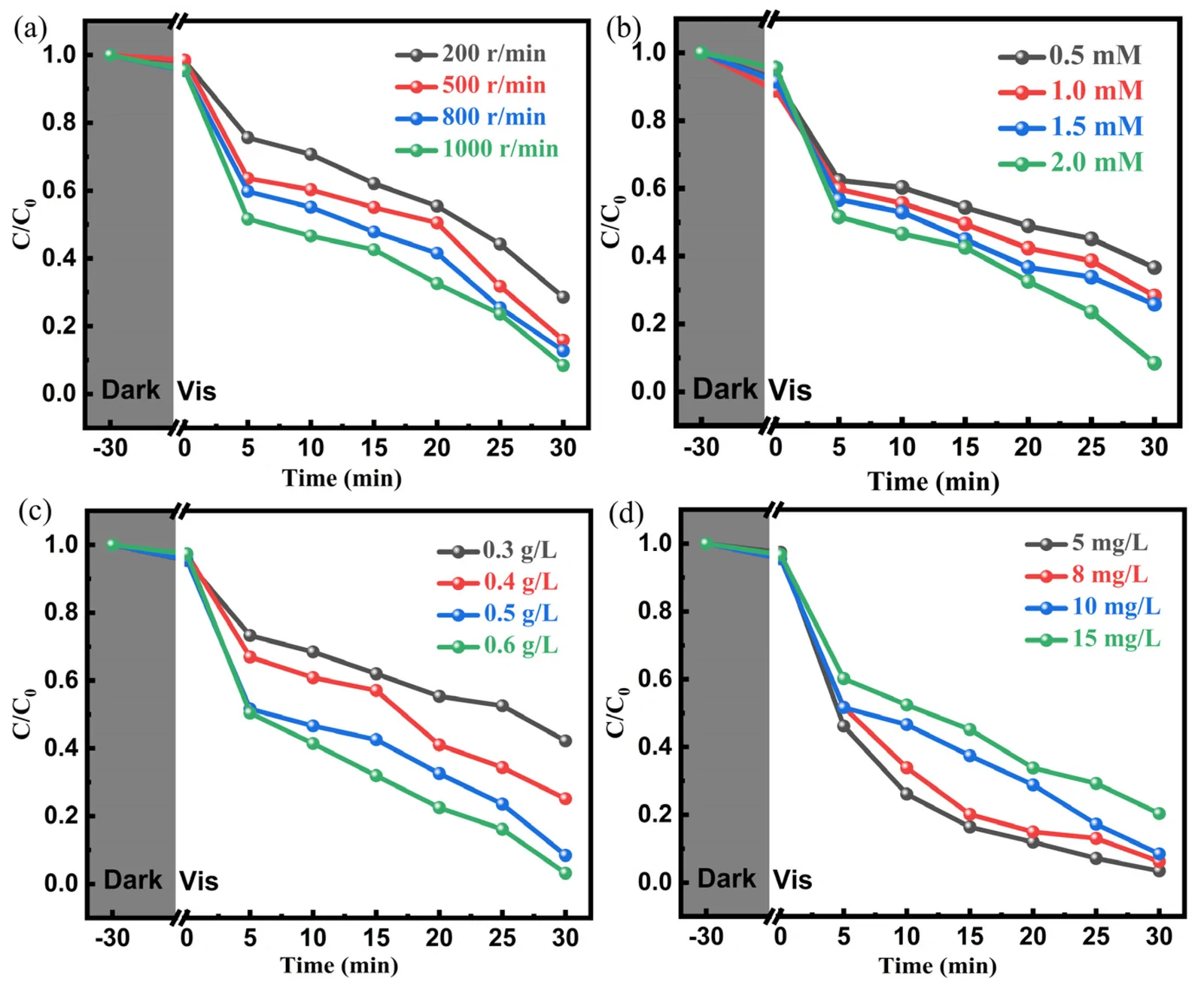
4-CP removal under the operating parameters of rotation speed (**a**), PMS concentration (**b**), BM(1:3) dosage (**c**), and 4-CP dosage (**d**). Conditions: Rotation speed = 1000 r/min, PMS = 2.0 mM, 4-CP = 10 mg/L, catalyst = 0.5 g/L, initial pH = 4.7 (Unless otherwise indicated in the figure).

Figure 2 shows the influence of operating factors on 4-CP reduction. Stirring acts on the piezoelectric material by applying an external force to cause deformation, thereby generating the piezoelectric effect [26]. When the rotation speed was set at 200, 500, 800, and 1000 r/min, the corresponding removal efficiencies of 4-CP were 71.5%, 84.2%, 87.3%, and 91.6%, respectively, exhibiting a rising trend with increasing rotation speed. In general, the higher the rotation speed, the greater the degree of material deformation and the stronger the induced electric field [27], which ultimately results in an increase in the 4-CP removal rate (Figure 2a). Figure 2b illustrates that with PMS concentration increasing from 0.5 to 2.0 mM, the 4-CP removal efficiencies increased from 63.4% to 91.6%. As the key oxidant, PMS concentration directly affects the production of ROS (such as SO_4_•^−^ and •OH), thereby determining the rate of pollutant oxidation [28]. With increasing BM(1:3) dosage (0.3, 0.4, 0.5, and 0.6 g/L), the corresponding 4-CP removal efficiencies were 57.9%, 75.0%, 91.6%, and 96.9%, respectively (Figure 2c). In general, the removal efficiencies improved with increasing catalyst dosage within the tested range. As the carrier of active sites, the catalyst dosage directly affects PMS activation and ROS generation, thereby determining the oxidation ability of the system toward pollutants [29]. With increasing initial 4-CP concentration (5, 8, 10, and 15 mg/L), the removal efficiencies were 96.6%, 93.9%, 91.6%, and 79.7%, respectively, showing a declining trend (Figure 2d). This decline indicates that at higher pollutant concentrations, the quantity of active sites accessible for oxidation per unit time becomes relatively insufficient, resulting in a lower removal efficiency [30].

**Figure 3:**
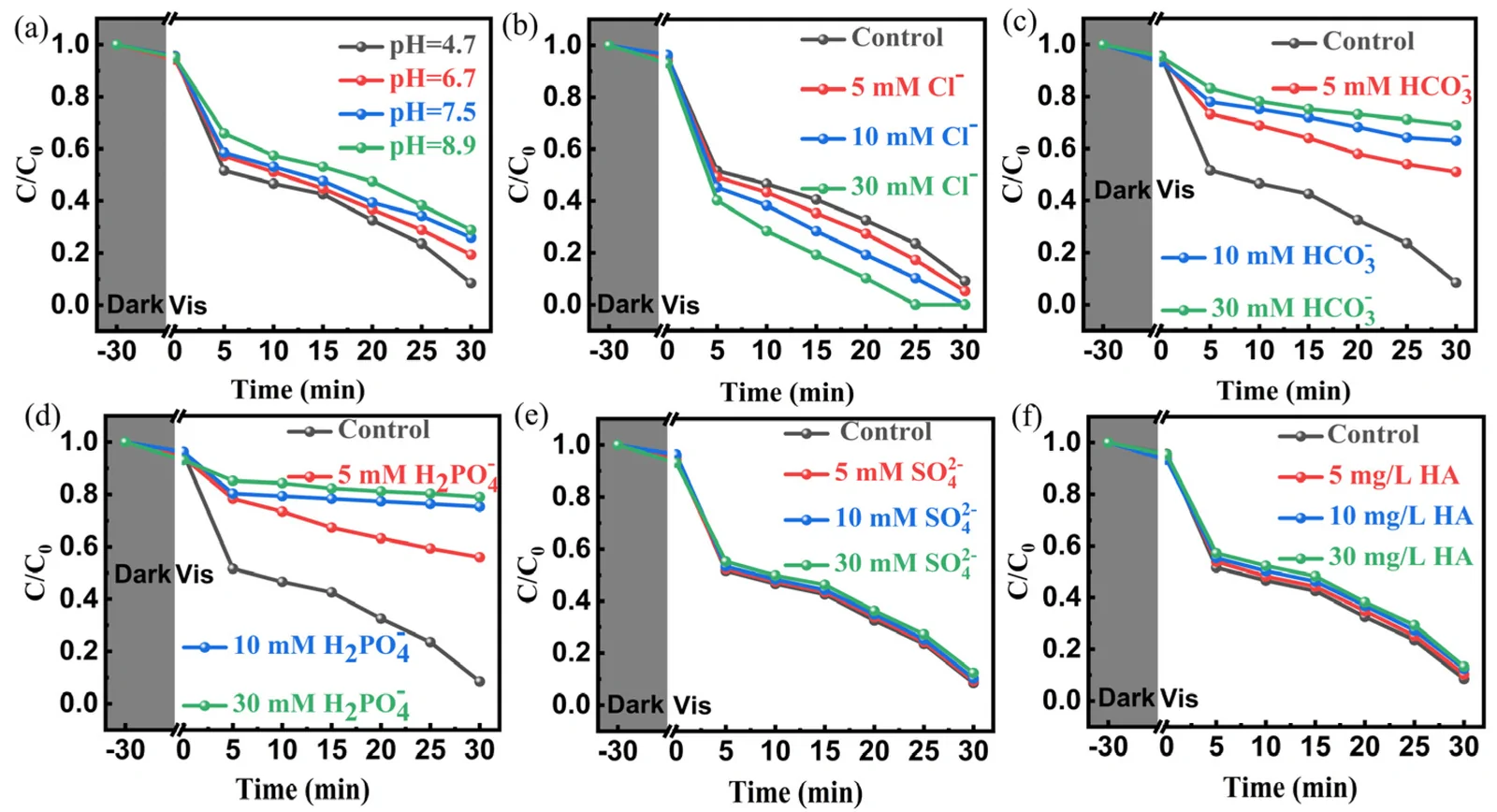
4-CP removal under different water quality conditions of pH (**a**), Cl^−^ concentration (**b**), HCO_3_^−^ concentration (**c**), H_2_PO_4_^−^ concentration (**d**), SO_4_^2−^ concentration (**e**), and HA concentration (**f**). Conditions: Rotation speed = 1000 r/min, [PMS] = 2.0 mM, [4-CP] = 10 mg/L, [catalyst] = 0.5 g/L, initial pH = 4.7 (Unless otherwise indicated in the figure).

Figure 3 presents the effect of various water quality parameters on 4-CP removal by the BM(1:3)/PMS/Vis–MS process. Figure 3a shows that the 4-CP removal rate varied considerably with pH. The highest value (91.6%) was observed at pH 4.7 within 30 min, which gradually decreased to approximately 81.7% at pH 6.7, 74.1% at pH 7.5, and 71.2% at pH 8.9. This trend suggests that acidic to neutral conditions favor the system’s catalytic performance, whereas alkaline media suppress its activity. As illustrated in Figure 3b, Cl^−^ concentrations in the range of 5–30 mM promoted 4-CP removal. Specifically, the promotion effect increased with increasing Cl^−^ concentration. Complete removal of 4-CP occurred within 30 min at 10 mM Cl^−^, and this time was shortened to 25 min when the Cl^−^ concentration reached 30 mM. This enhancement is attributed to the reaction between PMS and excess Cl^−^, which generates highly oxidizing species such as Cl_2_ and HClO, thereby facilitating the degradation of 4-CP [31]. Figure 3c,d shows that HCO_3_^−^ and H_2_PO_4_^−^ inhibited the degradation of 4-CP, and this inhibitory effect intensified with increasing ion concentration. The strong inhibitory effect of HCO_3_^−^ arises from two aspects: first, the alkaline environment resulting from HCO_3_^−^ hydrolysis; second, the consumption of •OH and SO_4_•^−^ by excess HCO_3_^−^, which converts them into the weakly oxidizing species CO_3_•^−^/HCO_3_•. H_2_PO_4_^−^ can also react with •OH and SO_4_•^−^ to generate the H_2_PO_4_•, which has low oxidizing power [32]. In addition, H_2_PO_4_^−^ is a negatively charged multidentate ligand that can adsorb strongly onto the catalyst surface, occupying active sites and preventing PMS or 4-CP molecules from approaching the catalytic center, thereby reducing the activation efficiency of PMS [33]. SO_4_^2−^ has no significant influence on 4-CP reduction within the system (Figure 3e). At concentrations of 5–30 mg/L, HA caused a minor decline in 4-CP degradation (with removal efficiency decreasing from 91.2% to 86.7%), which is negligible (Figure 3f). This marginal suppression is ascribed to the presence of abundant functional groups in HA, including phenolic hydroxyl and carboxyl moieties, which can rapidly scavenge SO_4_•^−^ and •OH [34]. This suggests that the BM(1:3)/PMS/Vis-MS process exhibits good resistance to HA interference. This robustness may be attributed to the composite material’s high specific surface area and numerous active sites, allowing it, to a certain degree, to counteract the competitive adsorption by HA [35]; in addition, non-radical pathways may also exist in the system, reducing its dependence on radicals and thereby enhancing its stability.

### 2.3. Trapping of ROS

**Figure 4:**
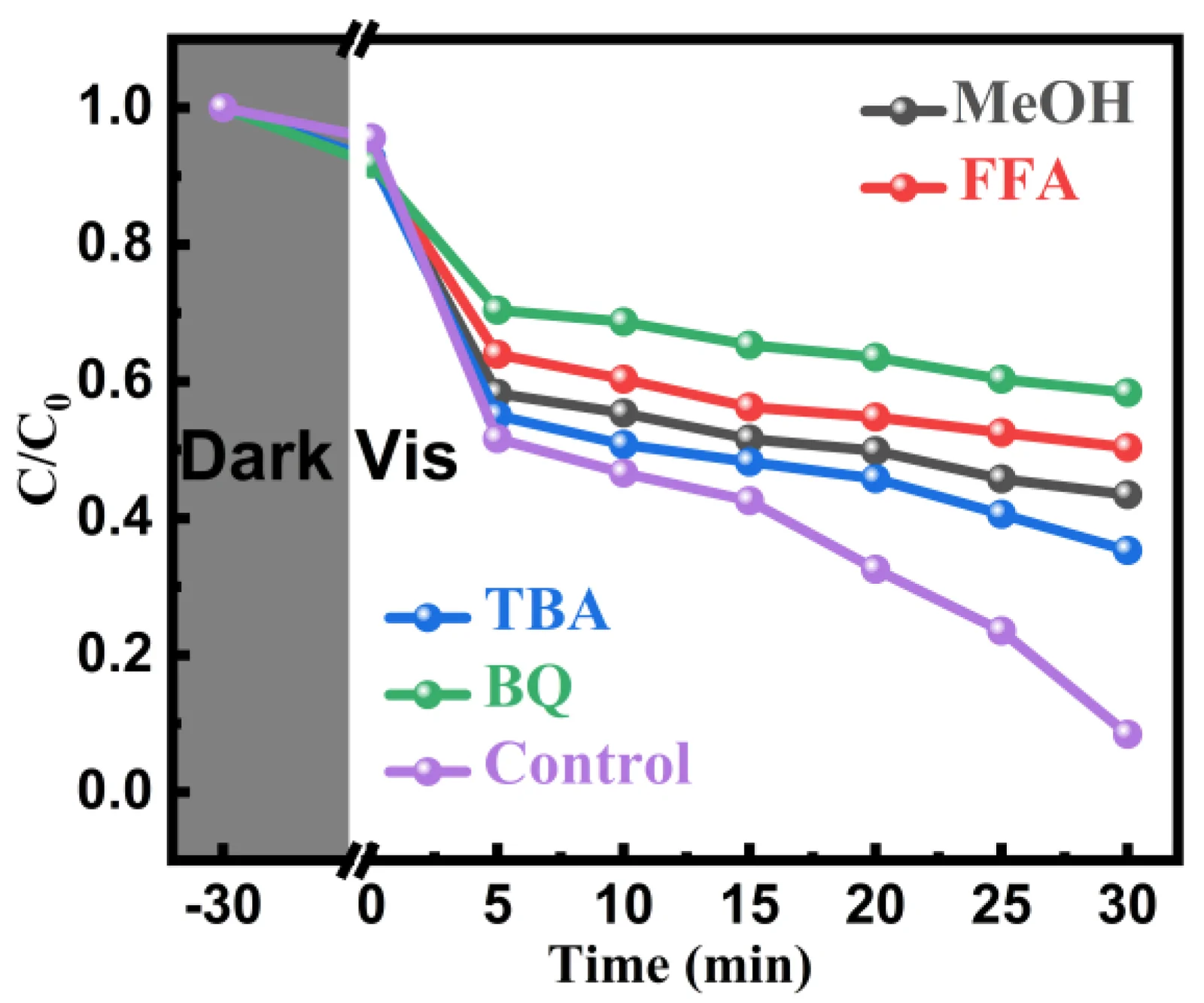
The effect of quenchers. Conditions: Rotation speed = 1000 r/min, PMS = 2.0 mM, 4-CP = 10 mg/L, BM(1:3) = 0.5 g/L, initial pH = 4.7, BQ = 0.5 mM, FFA = 1.0 mM, MeOH = 10 mM, TBA = 10 mM.

**Figure 5:**
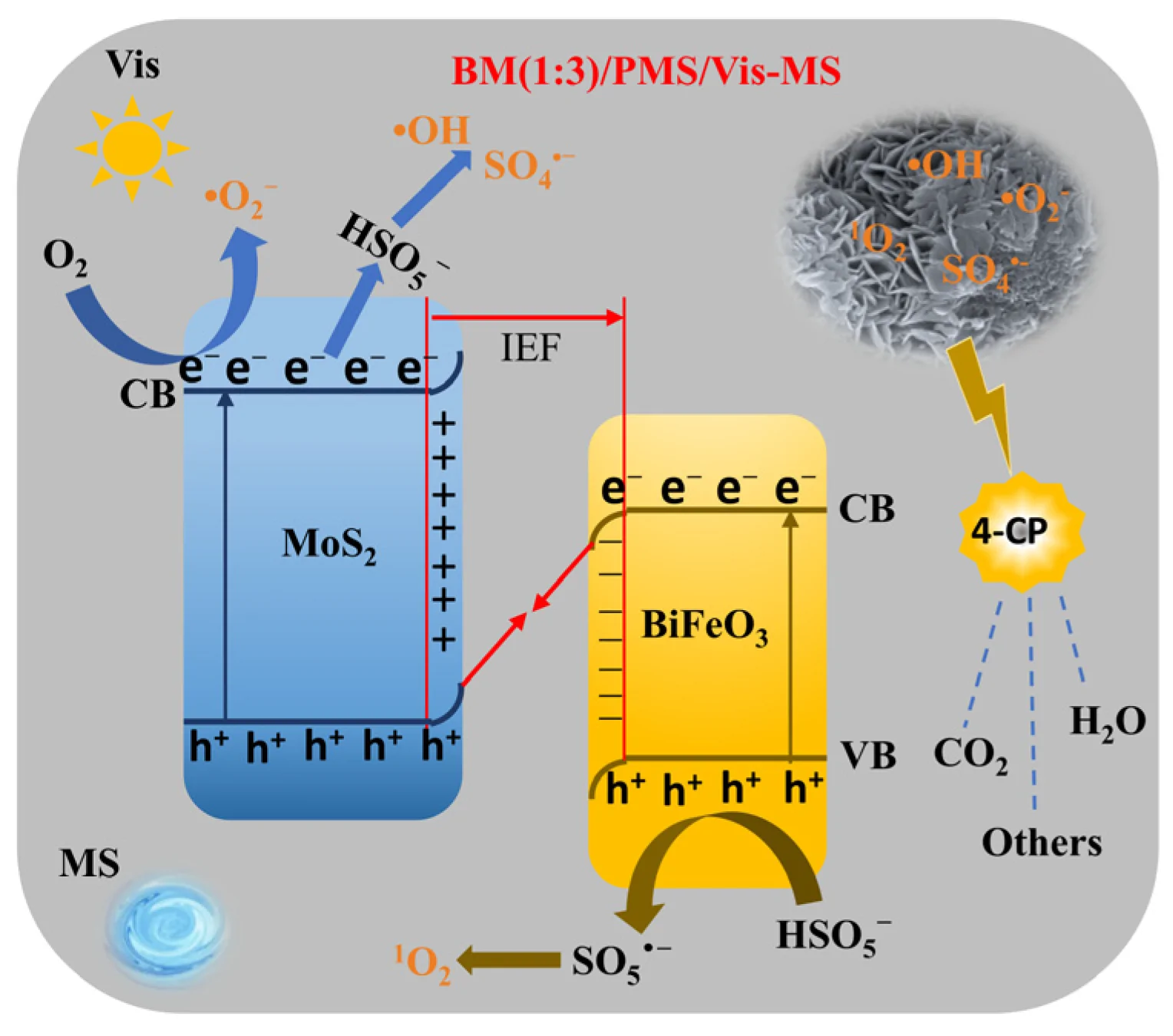
Mechanism of PMS activation by BM (1:3) for 4-CP degradation.

To determine the main ROS involved in the reaction process, radical trapping experiments were performed. p-benzoquinone (BQ) and furfuryl alcohol (FFA) were employed to probe the roles of superoxide radicals (•O_2_^−^) and ^1^O_2_, respectively [36]. Methanol (MeOH) was used as a broad-spectrum scavenger for quenching both •OH and SO_4_•^−^, while tert-butyl alcohol (TBA) was used as a selective scavenger targeting •OH [36]. As shown in Figure 4, upon adding BQ and FFA, the 4-CP removal rate dropped from 91.2% (without quenchers) to 41.6% and 49.7%, respectively, confirming that •O_2_^−^ and ^1^O_2_ were generated in the system. With the addition of TBA, the removal efficiency decreased to 64.7%, indicating that •OH were generated. Upon addition of MeOH, the removal rate further decreased to 57.6%, indicating that SO_4_•^−^ were also generated, albeit to a lesser extent than •OH. Overall, •O_2_^−^ were the most abundant reactive species generated in the system, followed by ^1^O_2_, •OH, and SO_4_•^−^. Figure 5 presents the mechanism diagram illustrating the process of BM(1:3)-activated PMS degradation of 4-CP. Under Vis irradiation, both MoS_2_ and BiFeO_3_ generate photogenerated electrons and holes. Meanwhile, under MS, an internal electric field (IEF) is generated within MoS_2_ and BiFeO_3_. According to our previous study [19], the conduction band (CB) and valence band (VB) positions of MoS_2_ are situated at −0.41 eV and 2.28 eV, respectively, whereas those of BiFeO_3_ are located at 0.31 eV and 2.54 eV, respectively. Under the piezoelectric field, the holes in the VB of MoS_2_ tend to recombine with the electrons in the CB of BiFeO_3_. This process suppresses the recombination of the electrons in the CB of MoS_2_ with the holes in the VB of BiFeO_3_. The key reactions involved in the generation of h^+^, e^−^, •O_2_^−^, ^1^O_2_, •OH, and SO_4_•^−^ are summarized in Equations (1)–(10).

(1) $${\mathrm{BiFeO}}_{3}{/\mathrm{MoS}}_{2}+\mathrm{hv}\;(\mathrm{visible}\;\mathrm{light})\to h^{+}+e^{-}$$

(2) $$\begin{array}{l} {\mathrm{BiFeO}}_{3}{/\mathrm{MoS}}_{2}+\mathrm{mechanical}\;\mathrm{stress}\to \mathrm{piezoelectric}\;\mathrm{field}\to \\ {\mathrm{enhance}\;\mathrm{the}\;\mathrm{separation}\;\mathrm{of}\;e}^{-}{/h}^{+} \end{array}$$

(3) $$e^{-}+{\mathrm{HSO}}_{5}^{-}\to {\mathrm{SO}}_{4}^{•-}+{\mathrm{OH}}^{-}$$

(4) $$h^{+}+{\mathrm{HSO}}_{5}^{-}\to {\mathrm{SO}}_{5}^{•-}+H^{+}$$

(5) $${\mathrm{SO}}_{4}^{•-}+H_{2}O\to {\mathrm{SO}}_{4}^{2-}+•\mathrm{OH}+H^{+}$$

(6) $$O_{2}+e^{-}\to O_{2}^{•-}$$

(7) $$O_{2}^{•-}+e^{-}+{2H}_{2}O\to 2•\mathrm{OH}+{2\mathrm{OH}}^{-}$$

(8) O2•−+h+→O21

(9) O2•−+O2•−→O21+H2O2

(10) SO5•−+SO5•−→2SO42−+O21

### 2.4. Density Functional Theory Calculations of 4-CP

**Figure 6:**
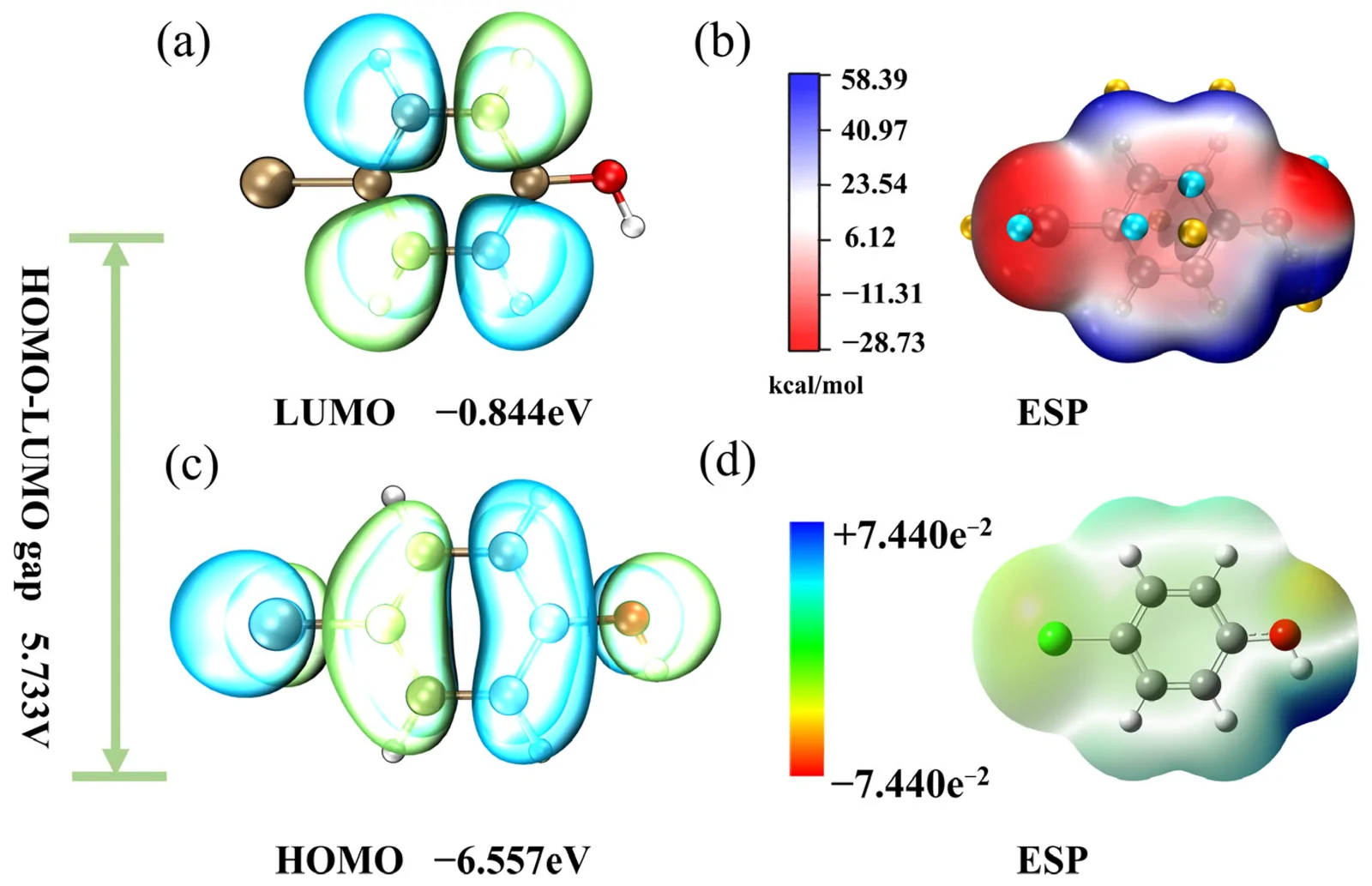
(**a**) LUMO, (**b**) ESP map with LUMO energy, (**c**) HOMO, and (**d**) ESP map with HOMO energy.

**Figure 7:**
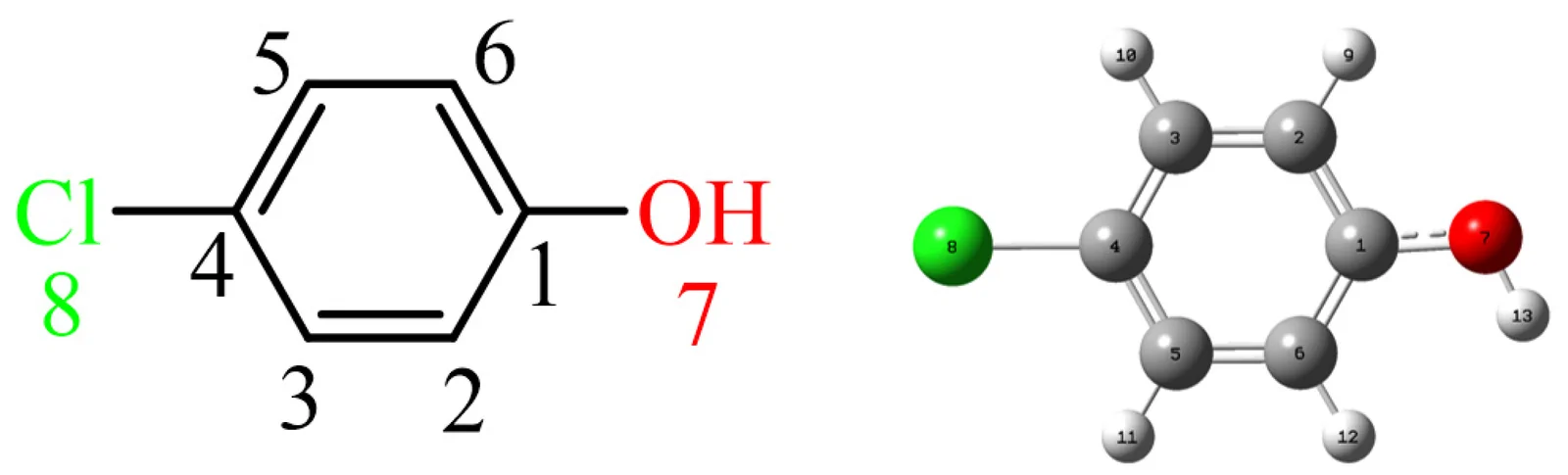
Optimized molecular structure of 4-CP with atomic numbering.

Density functional theory (DFT) calculations are widely recognized as a powerful tool for exploring the degradation mechanisms of organics. In this work, DFT was employed to characterize the molecular properties of 4-CP and to reveal potential degradation reaction mechanisms. Through electrostatic potential (ESP) calculations, the optimal nucleophilic and electrophilic reaction sites in the 4-CP molecule, along with the LUMO and HOMO orbitals, were identified. Figure 6a and Figure 6c illustrate the LUMO and HOMO orbitals of the 4-CP molecule, respectively, which have an energy gap of 5.733 eV. A lower energy gap indicates higher reactivity of the molecule. The HOMO is distributed throughout the entire 4-CP molecule, while the LUMO is primarily localized on the benzene ring. Figure 6b and Figure 6d show the optimized geometric structure and ESP map of 4-CP, respectively. In the ESP map, the red regions indicate electron-rich sites (negative electrostatic potential) that are prone to electrophilic attack; the blue regions denote electron-deficient sites (positive electrostatic potential) that are vulnerable to nucleophilic attack [37]. In addition, the Fukui indices (including f^+^ for nucleophilic attack, f^−^ for electrophilic attack, and f^0^ for radical attack) of the 4-CP molecule were determined (Table 1 and Figure 7) to forecast the radical-related degradation routes of 4-CP; in general, higher values indicate greater susceptibility of the corresponding site to the specific attack. The most vulnerable sites for radical attack (f^0^) are Cl (0.2102) and O (0.0893), suggesting that the C–Cl bond and the phenolic hydroxyl group are primary targets for radical-induced degradation. For nucleophilic attack (f^+^), Cl (0.1584) and C6 (0.1037) exhibit the highest values, while for electrophilic attack (f^−^), Cl (0.2619) and O (0.1314) are the most prominent sites.

### 2.5. Analysis of 4-CP Degradation Pathways

**Figure 8:**
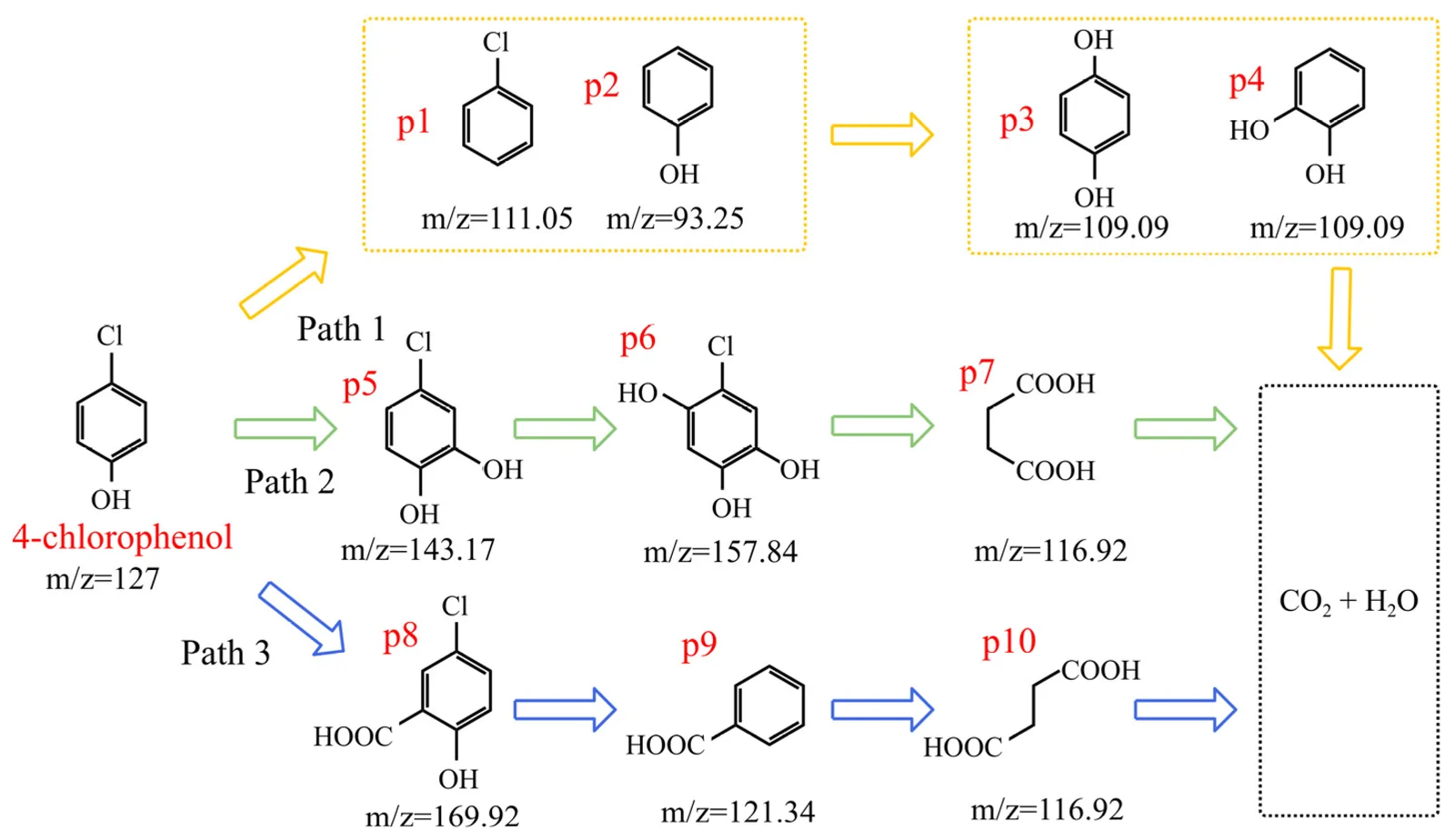
Possible degradation pathways of 4-CP.

The degradation pathways of 4-CP were deduced from the molecular weights and structural formulas of the intermediates identified, as depicted in Figure 8. In Pathway I, under the action of superoxide radicals and singlet oxygen, 4-CP is transformed into chlorobenzene (p1) and phenol (p2); subsequently, chlorobenzene is converted to phenol under the action of superoxide radicals, and phenol is further oxidized to o-hydroquinone (p4) and p-hydroquinone (p3). In Pathway II, superoxide radicals attack the ortho position relative to the hydroxyl group of p-chlorophenol, yielding p5; p5 undergoes further hydroxylation to form p6, which is then oxidized to p7 by singlet oxygen. In Pathway III, a carboxyl group is introduced adjacent to the hydroxyl group of 4-CP to generate p8; p8 then loses Cl^−^ and OH^−^ directly to form benzoic acid (p9), which is oxidized by singlet oxygen to form succinic acid (p10). Ultimately, these small-molecule intermediates undergo further mineralization to CO_2_ and H_2_O.

### 2.6. Assessment of Detoxification Ability

**Figure 9:**
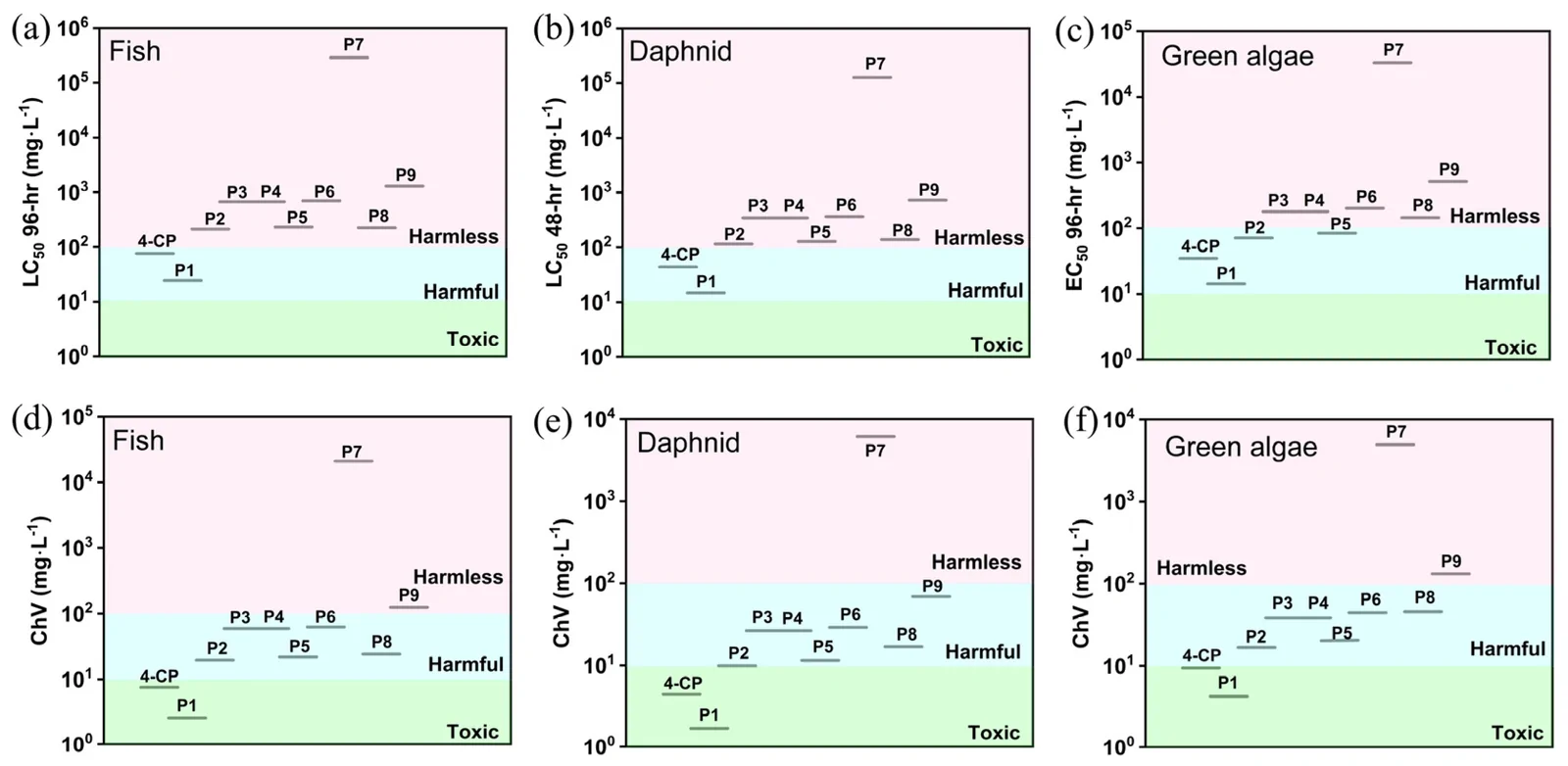
ECOSAR ecotoxicity estimates of 4-CP and its degradation products ((**a**–**c**) for acute toxicity, (**d**–**f**) for chronic toxicity).

The acute and chronic toxicity of 4-CP and its degradation products to fish, Daphnia, and green algae were estimated using the ECOSAR model, and the calculated results are presented in Table 2 and Figure 9. The estimated results reveal that, except for intermediate P1, the toxicity of the remaining degradation products was lower than that of the original compound 4-CP. Considering that the concentrations of the intermediate products during the degradation process were relatively low, the overall toxicity of the 4-CP solution was significantly reduced after oxidation treatment with the BM(1:3)/PMS/Vis-MS process.

### 2.7. Material Stability and Water Quality Compatibility

**Figure 10:**
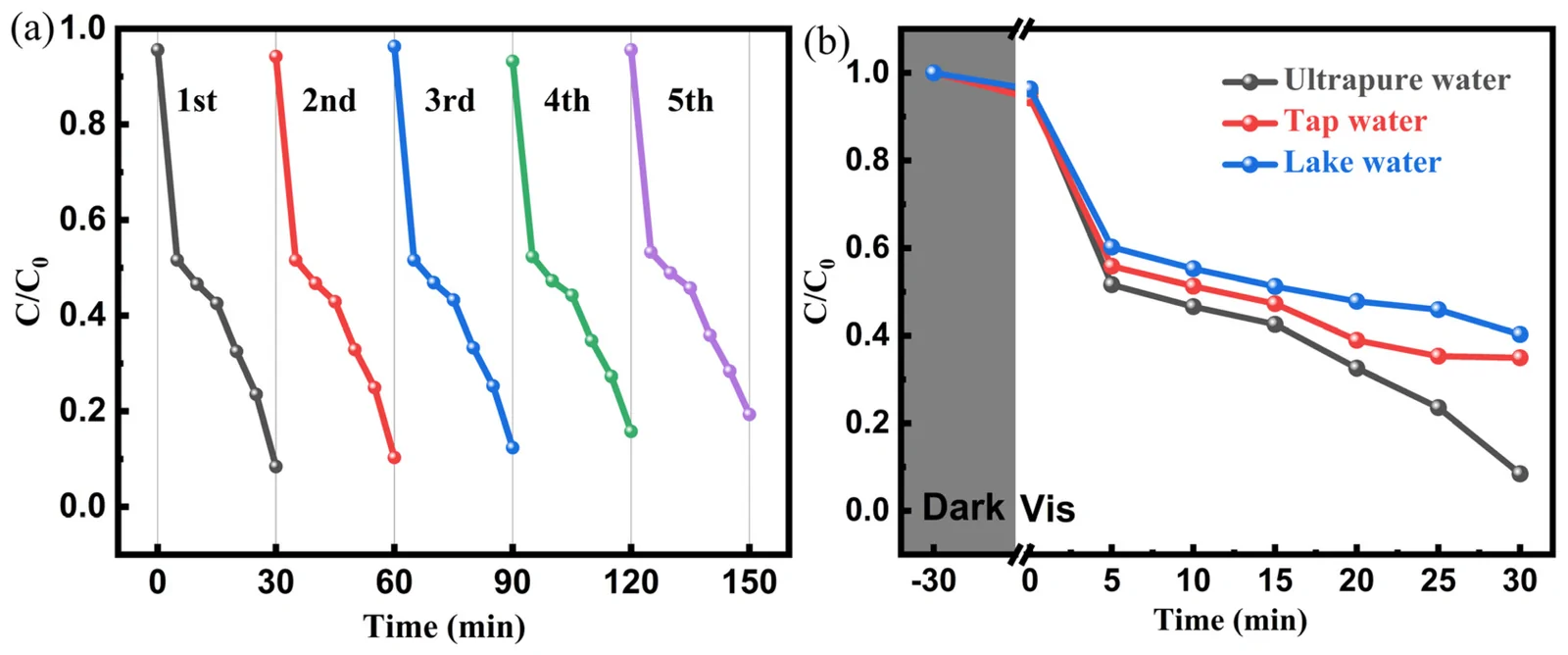
Reusability of the catalyst (**a**) and the effect of water matrices (**b**). Conditions: Rotation speed = 1000 r/min, PMS = 2.0 mM, 4-CP = 10 mg/L, BM(1:3) = 0.5 g/L, initial pH = 4.7.

After each reaction, the BM (1:3) catalyst was collected by centrifugation, washing, and drying, and then reused in the next cycle. A total of five consecutive cycles were performed, and the corresponding results are displayed in Figure 10a. With increasing cycle number, the degradation efficiency of 4-CP exhibited a slight downward trend. This decline may be ascribed to the irreversible loss of some active sites on the catalyst surface during the catalytic process. In addition, repeated redox reactions may lead to lattice distortion, metal ion leaching, and nanoparticle agglomeration. In particular, for two-dimensional materials such as MoS_2_, interlayer stacking tends to occur, further reducing their effective specific surface area and the number of active sites exposed [38]. Additionally, prolonged visible light irradiation during operation may also induce photocorrosion effects; in particular, for oxide components containing transition metals (e.g., BiFeO_3_), this can easily lead to changes in metal valence states or even localized structural collapse [39]. Nevertheless, after five consecutive reaction runs, the BM(1:3)/PMS/Vis-MS process kept a removal rate exceeding 80% for 4-CP, clearly confirming the outstanding reusability of the composite catalyst.

As shown in Figure 10b, the BM(1:3)/PMS/Vis-MS catalytic system exhibited removal efficiencies of 98.2%, 66.2%, and 59.8% within 30 min for 4-CP solutions prepared with ultrapure water, tap water, and natural lake water, respectively. These results clearly demonstrate that, despite the complex composition of real-world water matrices, this catalytic system can still achieve efficient removal of 4-CP, showing good environmental adaptability and application potential. In the ultrapure water system, which contains virtually no coexisting ions, natural organic matter (NOM), or other interfering components, the active sites on the catalyst surface are fully exposed to and effectively utilized by PMS and the target pollutant. Meanwhile, the separation of photogenerated charge carriers remains unaffected by external factors; consequently, the system exhibits optimal catalytic performance. In contrast, real water samples (tap water and lake water) had a non-negligible inhibitory effect, decreasing the removal efficiency by approximately 32% compared to that in ultrapure water. This suppression is likely attributable to the competitive consumption of reactive radicals by coexisting inorganic anions and natural organic matter. Nevertheless, the system maintained a removal rate of ~60% for 4-CP in untreated natural water within 30 min, demonstrating its potential for practical deployment. Future optimization of the catalyst dosage or PMS concentration could further compensate for the matrix effects, enabling more robust performance in complex water environments.

## 3. Materials and Methods

### 3.1. Chemicals

The chemicals involved in the experiments, along with their molecular formulas and manufacturers, are listed in Table 3. All reaction solutions were prepared using deionized water with the appropriate reagents.

### 3.2. Experimental Procedures

**Figure 11:**
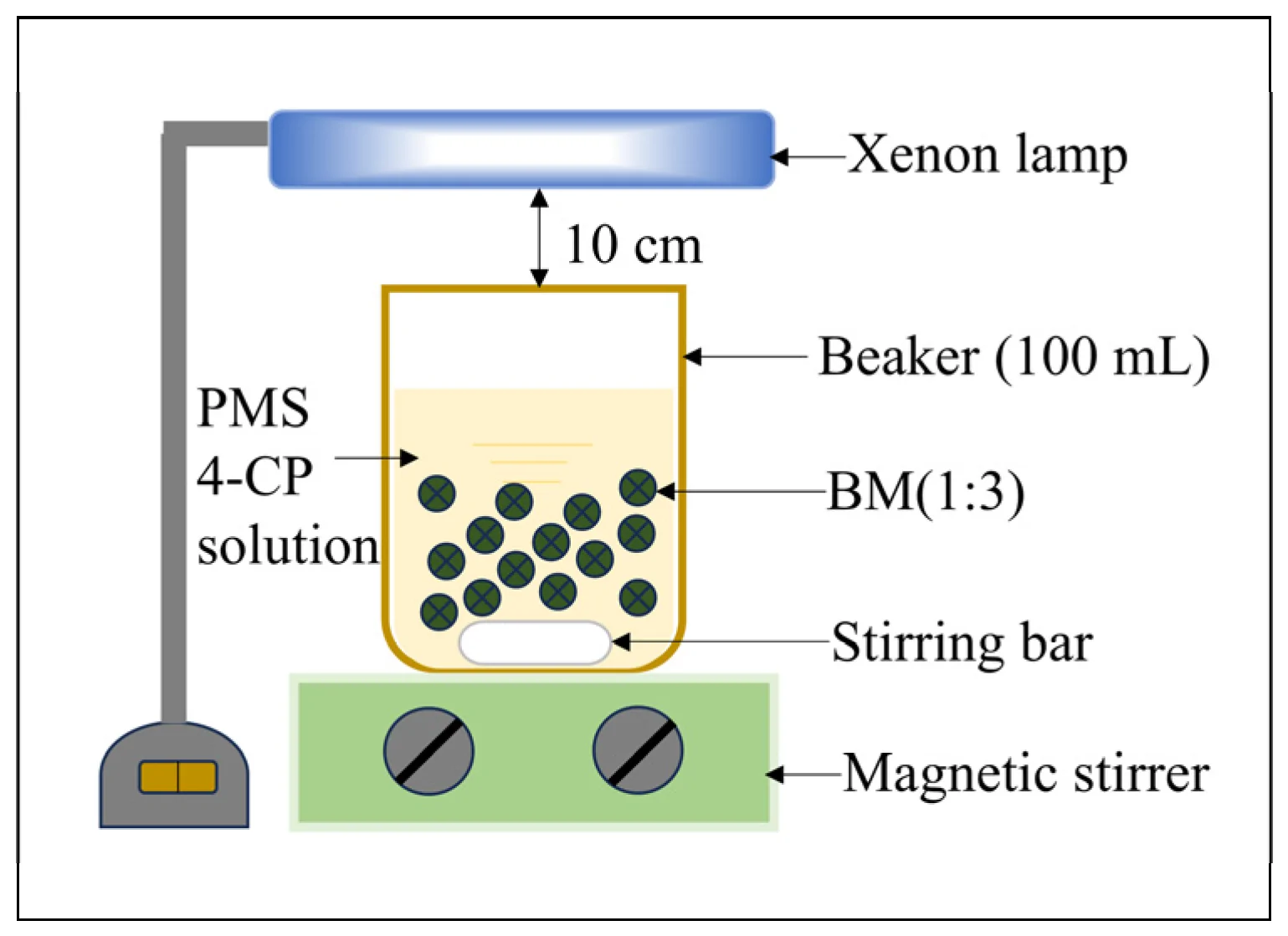
Schematic diagram of the reaction setup.

The various BiFeO_3_/MoS_2_ catalysts used in this study were synthesized in our earlier work and characterized in our previous publication [19]. The catalysts BM(2:1), BM(1:1), BM(1:2), and BM(1:3) represent different mass ratios of BiFeO_3_ to MoS_2_, as described in Song et al. [19]_._ All experiments were performed in a 100 mL beaker, equipped with a xenon lamp (PLS-SXE300+, Beijing Perfectlight Co., Ltd., Beijing, China) providing photocatalytic conditions and a magnetic stirrer (90-2, Changzhou Jintan Kexing Instrument Factory, Changzhou, China) serving as the piezoelectric excitation source. The entire reaction apparatus was placed in a closed chamber. The schematic diagram of the reaction setup is shown in Figure 11. Before the reaction, the catalyst was dispersed in 50 mL of a 4-CP solution at a concentration of 10 mg/L, and the suspension was stirred in the dark for 30 min to reach adsorption equilibrium. Subsequently, a given amount of PMS was added, and the light illumination and stirring were started simultaneously to trigger the degradation reaction. At each preset time point, samples were collected, and the reaction was terminated by adding sodium thiosulfate. After filtration through a 0.22 μm filter, the samples were subjected to analysis using high-performance liquid chromatography (HPLC). The initial pH of the solution was adjusted using 0.1 M H_2_SO_4_ or NaOH. To investigate the influence of water quality, NaCl, Na_2_SO_4_, NaHCO_3_, NaH_2_PO_4_ and humic acid (HA) were separately added to study the influence of Cl^−^, SO_4_^2−^, HCO_3_^−^, H_2_PO_4_^−^, and HA on the degradation efficiency. In the trapping experiment, MeOH, TBA, BQ, and FFA were introduced before the reaction, and the remaining procedures were consistent with those in the degradation test. All experiments were carried out in triplicate.

### 3.3. Analytical Methods

The 4-CP concentration was measured by HPLC (Agilent 1260, Santa Clara, CA, USA) using an Agilent ZORBAX SB-C18 column (4.6 mm × 250 mm, 5 μm) maintained at a temperature of 30 ± 1 °C. The mobile phase was composed of 0.1% formic acid in water and methanol (32:68, *v*/*v*) delivered at a flow rate of 0.8 mL/min. The injection volume was set at 50 μL, and the detection wavelength was set to 224 nm. Under these conditions, the elution time of the 4-CP was approximately 4.3 min. The reaction intermediates were identified via liquid chromatography–mass spectrometry (LC-MS, Agilent 1290uplc/qtof6550, USA) using a reversed-phase C18 column in ESI^+^ mode, operating over a scan range of *m*/*z* 50–500. The mobile phase consisted of acetonitrile (A) and 0.1% formic acid in water (B) at a column temperature of 30 °C and a flow rate of 0.12 mL/min. The gradient elution program was as follows: 15% A (0–5 min), 15% to 85% A (5–10 min), 85% A (10–20 min), and 85% to 15% A (20–30 min). Prior to analysis, all samples were purified using an MCX solid-phase extraction column. The mineralization rate was measured by a total organic carbon (TOC) analyzer (Vario TOC cube, Elementar, Langenselbold, Germany).

## 4. Conclusions

In this study, a piezophotocatalytic material, BiFeO_3_/MoS_2_, was employed to catalyze PMS for 4-CP removal. The performance of materials with various BiFeO_3_-to-MoS_2_ ratios toward 4-CP removal was investigated. Key influencing factors on 4-CP degradation in the BM(1:3)/PMS system were examined, and the underlying mechanism was analyzed. Moreover, the toxicity of the degradation intermediates was assessed. Based on these findings, the following conclusions were drawn.

1. BM(1:3)/PMS process was superior to the BM(2:1)/PMS, BM(1:1)/PMS, and BM(1:2)/PMS processes in removing 4-CP, and it could remove 91.6% of 4-CP within 30 min.
2. The rotation speed, PMS concentration, BM(1:3) dosage, and initial 4-CP concentration all had significant effects on 4-CP reduction by BM(1:3)-activated PMS. The first three factors were positively correlated with the 4-CP degradation rate, while the last one was negatively correlated with it. A lower pH value was more favorable for 4-CP removal. Chloride ions promoted the degradation of 4-CP, whereas bicarbonate (HCO_3_^−^) and dihydrogen phosphate (H_2_PO_4_^−^) inhibited the removal of 4-CP. Sulfate ions (SO_4_^2−^) and humic acid (HA) exhibited negligible effects.
3. The 4-CP degradation was mainly driven by the synergistic effects of H^+^, •O_2_^−^, ^1^O_2_, •OH, and SO_4_•^−^ generated in the system. Furthermore, Fukui function analysis identified the vulnerable sites on 4-CP susceptible to attack.
4. 4-CP was degraded via three pathways to yield P3, P4, P7, P10, as well as CO_2_ and H_2_O. With the exception of P1, all other degradation products exhibited lower toxicity than 4-CP.
5. After five consecutive recycling runs, the 4-CP removal achieved by BM(1:3)-activated PMS decreased from 91.6% to 80.7%. Moreover, the degradation rate in the lake water matrix dropped significantly, from 91.6% to 59.8%.

This study provides a valuable reference for the removal of refractory organics from water via BM(1:3) material to activate PMS. In the future, more attention should be paid to the optimization of operational parameters to enhance the degradation performance of this material toward organic pollutants in actual water bodies.

## Figures and Tables

**Table 1:** Atomic charges and condensed Fukui functions of 4-CP.

	Atomic Numbering	N (Neutral)	q (N + 1)	q (N − 1)	f^+^ (for Nucleophilic Attack)	f^−^ (for Electrophilic Attack)	f^0^ (for Radical Attack)
C	1	0.2509	0.2494	0.2759	0.0015	0.0251	0.0133
C	2	−0.1544	−0.2537	−0.0925	0.0993	0.0619	0.0806
C	3	−0.0359	−0.1298	0.0037	0.0940	0.0396	0.0668
C	4	−0.3106	−0.2709	−0.3015	−0.0397	0.0091	−0.0153
C	5	−0.0423	−0.1421	0.0016	0.0998	0.0438	0.0718
C	6	−0.1584	−0.2620	−0.0925	0.1037	0.0658	0.0848
O	7	−0.6161	−0.6632	−0.4847	0.0471	0.1314	0.0893
Cl	8	−0.0278	−0.1862	0.2341	0.1584	0.2619	0.2102
H	9	0.1824	0.0808	0.2617	0.1016	0.0793	0.0905
H	10	0.1877	0.0907	0.2600	0.0970	0.0723	0.0847
H	11	0.1870	0.0901	0.2605	0.0969	0.0736	0.0852
H	12	0.1640	0.0626	0.2426	0.1014	0.0786	0.0900
H	13	0.3734	0.3343	0.4310	0.0390	0.0576	0.0483

**Table 2:** Toxicity calculation results.

Name	Acute Toxicity			Chronic Toxicity		
	Fish (96 h) LC50	Daphnid (48 h) LC50	Green Algae (96 h) EC50	Fish	Daphnid	Green Algae
4-CP	76.3	43.9	34.8	7.59	4.46	9.40
P1	24.3	14.9	14.2	2.59	1.71	4.23
P2	212	115	71.1	19.6	9.89	16.8
P3	669	347	179	58.8	26.4	38.4
P4	669	347	179	58.8	26.4	38.4
P5	232	128	84	21.9	11.5	20.6
P6	694	366	201	62.2	29	44.5
P7	289,000	127,000	33,100	20,900	6110	4920
P8	225	139	145	24.3	17	45.7
P9	1300	730	518	125	68.9	132

**Table 3:** Chemicals used in this study.

Chemical	Chemical Formula	Manufacturer
4-chlorophenol	C_6_H_5_ClO	Shanghai Aladdin Biochemical Technology Co., Ltd. (Shanghai, China)
Potassium peroxymonosulfate	KHSO_5_·0.5KHSO_4_·0.5K_2_SO_4_	Shanghai Macklin Biochemical Co., Ltd. (Shanghai, China)
Humic acid	-	
p-Benzoquinone	C_6_H_4_O_2_	
Sodium thiosulfate	Na_2_S_2_O_3_	Sinopharm Chemical Reagent Co., Ltd. (Shanghai, China)
Sodium hydroxide	NaOH	
Sodium chloride	NaCl	
Sodium dihydrogen phosphate	NaH_2_PO_4_	
Sulfuric acid	H_2_SO_4_	
Anhydrous sodium sulfate	Na_2_SO_4_	
Anhydrous ethanol	C_2_H_6_O	
Sodium bicarbonate	NaHCO_3_	
Methanol	CH_4_O	
Furfuryl alcohol	C_5_H_6_O_2_	
tert-Butyl alcohol	C_4_H_10_O	

## Data Availability

Data are contained within the article.
